# Precision cancer therapy: profiting from tumor specific defects in the DNA damage tolerance system

**DOI:** 10.18632/oncotarget.24777

**Published:** 2018-04-10

**Authors:** Olimpia Alessandra Buoninfante, Bas Pilzecker, Muhammad Assad Aslam, Ioannis Zavrakidis, Rianne van der Wiel, Marieke van de Ven, Paul C.M. van den Berk, Heinz Jacobs

**Affiliations:** ^1^ Division of Tumor Biology and Immunology, Amsterdam, CX 1066, The Netherlands; ^2^ Division of Psychosocial Research and Epidemiology, Amsterdam, CX 1066, The Netherlands; ^3^ Mouse Clinic for Cancer and Aging research (MCCA) Transgenic Facility, The Netherlands Cancer Institute, Amsterdam, CX 1066, The Netherlands

**Keywords:** DNA damage tolerance, precision cancer medicine, cancer target, chemotherapy, cisplatin

## Abstract

DNA damage tolerance (DDT) enables replication to continue in the presence of a damaged template and constitutes a key step in DNA interstrand crosslink repair. In this way DDT minimizes replication stress inflicted by a wide range of endogenous and exogenous agents, and provides a critical first line defense against alkylating and platinating chemotherapeutics. Effective DDT strongly depends on damage-induced, site-specific PCNA-ubiquitination at Lysine (K) 164 by the E2/E3 complex (RAD6/18). A survey of The Cancer Genome Atlas (TCGA) revealed a high frequency of tumors presents RAD6/RAD18 bi-allelic inactivating deletions. For instance, 11% of renal cell carcinoma and 5% of pancreatic tumors have inactivating *RAD18*-deletions and 7% of malignant peripheral nerve sheath tumors lack *RAD6B*. To determine the potential benefit for tumor-specific DDT defects, we followed a genetic approach by establishing unique sets of DDT-proficient *Pcna*^*K164*^ and -defective *Pcna*^*K164R*^ lymphoma and breast cancer cell lines. In the absence of exogenous DNA damage, *Pcna*^*K164R*^ tumors grew comparably to their *Pcna*^*K164*^ controls *in vitro* and *in vivo*. However, DDT-defective lymphomas and breast cancers were compared to their DDT-proficient controls hypersensitive to the chemotherapeutic drug cisplatin (CsPt), both *in vitro* and *in vivo.* CsPt strongly inhibited tumor growth and the overall survival of tumor bearing mice greatly improved in the DDT-defective condition. These insights open new therapeutic possibilities for precision cancer medicine with DNA damaging chemotherapeutics and optimize Next-Generation-Sequencing (NGS)-based cancer-diagnostics, -therapeutics, and -prognosis.

## INTRODUCTION

Each tumor has its specific genetic make-up which determines not only its strength but also its weakness in response to specific stressors. With the rise of NGS-based technology and diagnostics, tumor-specific mutations and related vulnerabilities can be identified at an unprecedented accuracy and speed. NGS enables the identification of crucial mutations and molecular defects that impair tumor fitness. Its speed and accuracy set the molecular basis of personalized cancer medicine, characterized by more specific and strategic intervention approaches. Prime examples of such vulnerabilities are mutations that give rise to tumor-specific neo-antigens, that offered a concrete therapeutic window for immunotherapy, or inactivating mutations of components involved in homology directed repair of DNA double strand breaks. One of the most successful example is represented by the loss of function or mutations in *BRCA1, or BRCA2,* in breast and ovarian cancers, which render these tumors but not the environment hypersensitive to PARP-inhibitors [1]. This promising strategy is known as synthetic lethality, and current research is oriented towards the identification of other synthetic lethal interactions. This concept, where the DNA damage response (DDR) status of a tumor dictates the intervention mode, holds great promises in treating cancer. These examples highlight the necessity to screen for other tumor-specific defects in the DDR network, identify new tumor specific vulnerabilities and provide more specific tumor-intervention strategies. To determine whether this concept also holds for the DNA damage tolerance (DDT) system, more bench to bedside research is required.

Within the DDR network, the capacity to tolerate rather than repair DNA lesions is an important contributor to genetic stability and cellular fitness [2, 3]. DDT enables replication to continue in the presence of a damaged template, thereby alleviating replication stress imposed by a wide variety of naturally occurring DNA lesions as well as base modifications inflicted by a variety of chemotherapeutic DNA-alkylating and -platinating agents. The capacity to tolerate replication blocking lesions and/or structures prevents prolonged replication fork stalling that can cause a fork collapse, and highly genotoxic DNA double strand breaks (DSBs) [4]. Therefore, inactivating mutations in the DDT system in tumors may render those hypersensitive to certain DNA lesions, induced by specific DNA damaging agents.

Within the DDT system four principal modes can be distinguished, translesion synthesis (TLS), template switching, fork reversal, and repriming. Moreover, if repriming behind the lesion takes place, subsequent TLS or template switching allows ‘post-replicative’ DNA synthesis opposite the lesion. While TLS is facilitated by damage-inducible site-specific mono-ubiquitination at lysine (K) 164 of the DNA-clamp PCNA (PCNA^K164^), template switching and fork reversal are facilitated by K63-linked poly-ubiquitination of the same K164 residue (PCNA-Ub^n^) [5, 6]. Damage-induced mono-ubiquitination of PCNA (PCNA-Ub), is mediated by the E2/E3 complex Rad6/Rad18 [4, 6–8] . PCNA-Ub recruits TLS polymerases through their PCNA interacting peptide box, ubiquitin-binding domain Ub-binding motif or the Ub-binding zinc finger [9, 10]. This combined affinity greatly facilitates its replacement with the high-fidelity replicative polymerase. In this way damage-induced PCNA-Ub serves as a molecular switch from damage-intolerant, proof-read active, replicative DNA polymerase D or E to one of the damage-tolerant, proof-read inactive, Y-family TLS polymerase, POLH, POLK, REV1, or POLI [11–14]. The unique capacity to accommodate non-Watson/Crick base pairs in their catalytic center enables direct replication opposite damaged templates. A wide range of DNA lesions, such as UV-induced cyclobutane pyrimidine dimers (CPD) and 6-4 photoproducts (6-4 PP), oxidized or alkylated DNA bases, non-instructive abasic sites, or unhooked interstrand crosslinks (ICLs) can be tolerated, the latter provides a key step in the repair of ICLs [13, 15]. Despite the lack of proof read activity, our genome-wide mutation studies suggest that overall TLS polymerases contribute to genome maintenance, and hence act anti-mutagenic [16]. Concomitantly, a single DDT defect can render the system hyper-sensitive and hyper-mutagenic to specific lesions, including those inflicted by established and widely applied chemotherapeutics. The resulting genomic instability promotes genetic heterogeneity, which is instrumental for tumor biology.

Screening TCGA for bi-allelic inactivating deletions in the DDT system indicated a high contribution of tumors with specific defects [17, 18]. Given the important role of the RAD6/RAD18 (E2/E3) complex in PCNA ubiquitination and polymerase switching from replicative DNA polymerases to damage tolerant TLS polymerases, there is an unmet need to explore if and how such defects can translate into precision cancer medicine. By generating sets of tumors proficient or deficient in PCNA ubiquitination, we put this concept to the test and provided concrete basis for future cancer intervention strategies. Our data indicate that a DDT defect can render tumors hypersensitive to existing widely applied DNA platinating agents like CsPt. Apparently, the DDT status of tumors is a critical predictor and determinant for tumor intervention with platinating agents, and relevant for personalized medicine.

## RESULTS

### Frequency of homozygous inactivating deletions in the DDT system in human tumors

**Figure 1:**
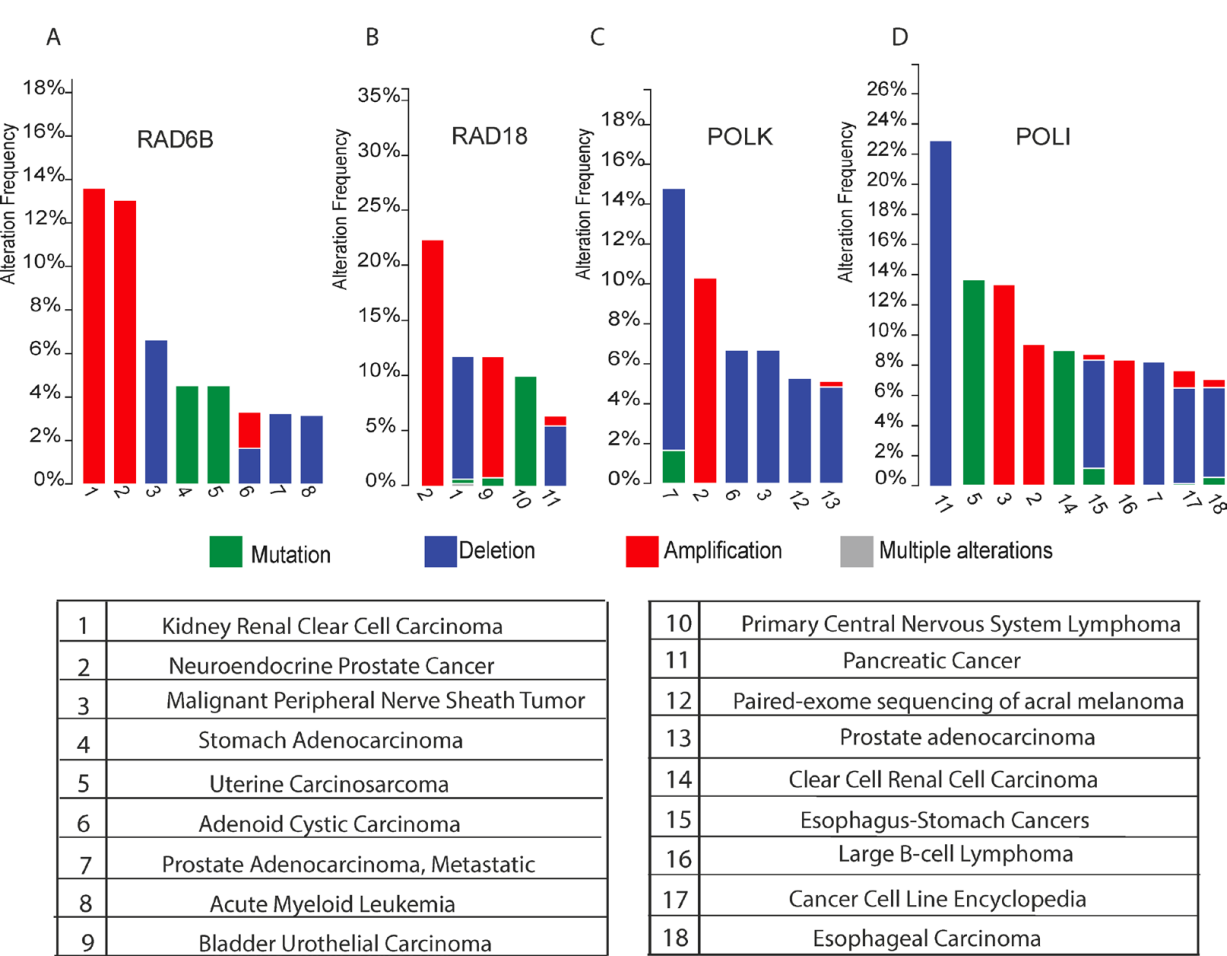
**(A–D)** Histograms show the cross-cancer alteration frequency of indicated genes (*RAD6B* Figure 1A), (*RAD18* Figure 1B), (*POLK* Figure 1C) and (*POLI* Figure 1D) for the indicated tumor types: 1 Kidney Renal Clear Cell Carcinoma (TCGA, Provisional); 2 Neuroendocrine Prostate Cancer (Trento/Cornell/Broad 2016); 3 Malignant Peripheral Nerve Sheath Tumor (MSKCC, Nat Genet 2014); 4 Stomach Adenocarcinoma (UHK, Nat Genet 2011); 5 Uterine Carcinosarcoma (Johns Hopkins University, Nat Commun 2014); 6 Adenoid Cystic Carcinoma (MSKCC, Nat Genet 2013); 7 Prostate Adenocarcinoma, Metastatic (Michigan, Nature 2012); 8 Acute Myeloid Leukemia (TCGA, Provisional); 9 Bladder Urothelial Carcinoma (TCGA, Nature 2014); 10 Primary Central Nervous System Lymphoma (Mayo Clinic, Clin Cancer Res 2015); 11 Pancreatic Cancer (UTSW, Nat Commun 2015); 12 Paired-exome sequencing of acral melanoma (TGEN, Genome Res 2017); 13 Prostate Adenocarcinoma (provisional); 14 Multiregion Sequencing of Clear Cell Renal Cell Carcinoma (IRC, Nat Genet 2014); 15 TCGA data for Esophagus-Stomach Cancers (TCGA, Nature 2017); 16 Lymphoid Neoplasm Diffuse Large B-cell Lymphoma (TCGA, Provisional); 17 Cancer Cell Line Encyclopedia (Novartis/Broad, Nature 2012); 18 Esophageal Carcinoma (TCGA, Provisional). The data were retrieved from cBioPortal [18, 17].

Tumors with specific defects in the DDR network offer great potential for intervention with specific DNA damaging agents. Present insights into DDT and the fact that TLS contributes as an essential intermediate step in ICL repair, strongly motivated us to further explore this therapeutic tactic. To estimate the fraction of patients with DDT-defective cancers we first determined the frequency of tumors with homozygous inactivating deletions in genes coding DDT components. A survey of ‘The Cancer Genome Atlas’ (TCGA) revealed a high frequency of tumors with DDT defects. For instance, 11% of renal cell carcinoma (RCC) and 5% of pancreatic tumors have inactivating RAD18-deletions and 7% of malignant peripheral nerve sheath tumors lack RAD6B. About 22% of pancreatic, 8% of prostate, 7% of esophagus tumors lack *POLI*, and up to 14% of prostate-cancers lack *POLK*. Homozygous inactivating deletions in REV1 are very infrequent, which likely relates to the critical non-catalytic activity of REV1 to recruit other TLS-polymerase members of the Y-family [19] (Figure 1A–1D).

Given the role of the Rad6/Rad18 (E2/E3) complex in PCNA ubiquitination to facilitate polymerase switching, we used tumors of genetically engineered mouse models carrying a non-modifiable *Pcna*^*K164R*^ mutant as well as a loxP flanked wild type *Pcna*^*flox*^ allele (*Pcna*^*flox*^*)* to investigate the impact of defective DDT on therapeutic outcome with CsPt.

### Establishing a DDT-proficient and defective-lymphoma model

**Figure 2:**
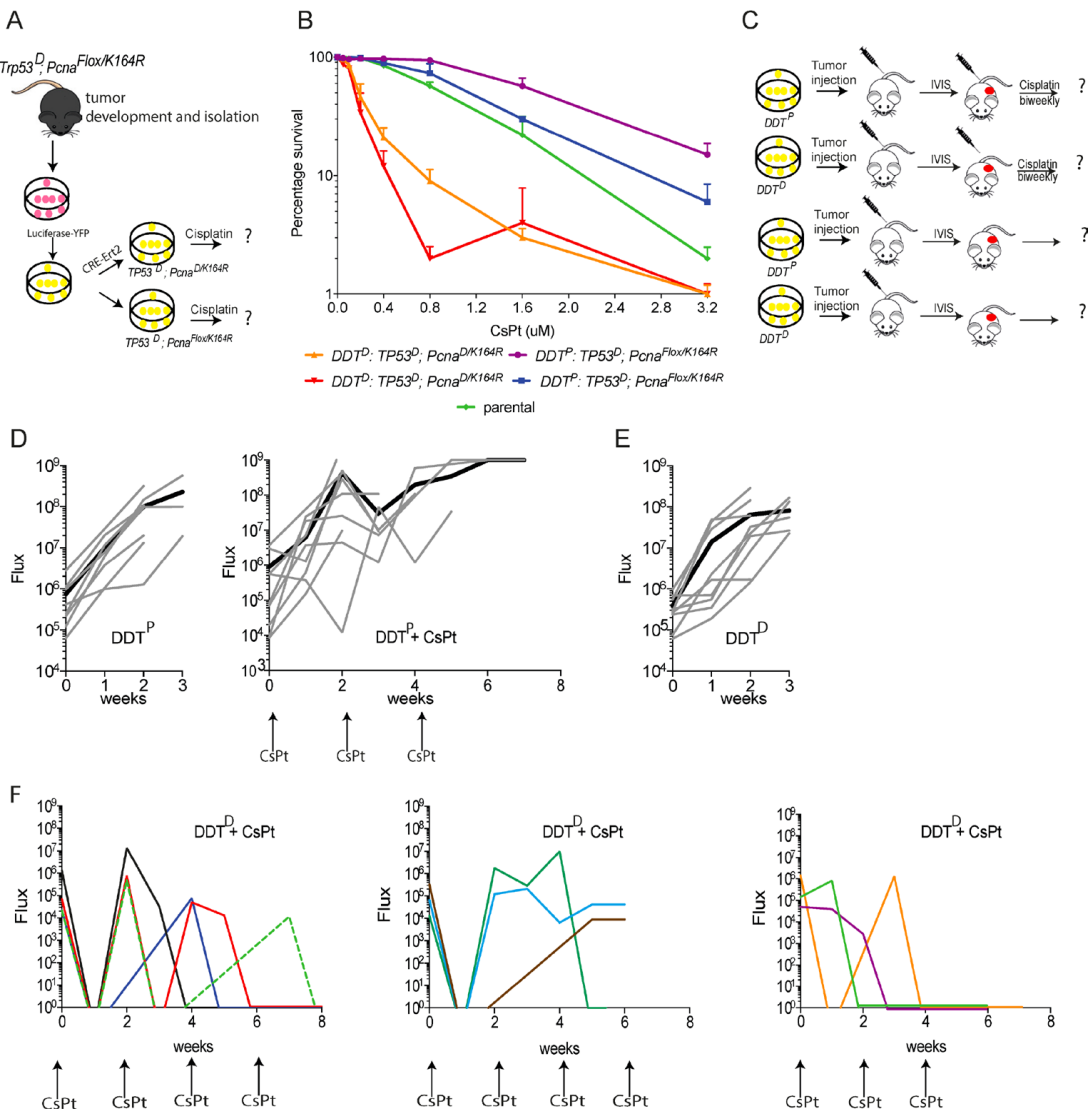
Assessing the chemosensitizing potential of lymphoma defective in PCNA ubiquitination **(A)** Schematic representation of the *in vitro* modification of lymphoma cells. **(B)** *In vitro* sensitivity assay of WT (DDT^P^) and PCNA^K164R^ mutant (DDT^D^) lymphomas to increasing CsPt doses. **(C)** Schematic representation of the *in vivo* approach. **(D)** *In vivo* bioluminescence imaging quantification as Flux (photons per second) of transplanted WT tumors (*n =* 8) control (left) or treated in response to 6 mg/kg CsPt treatment every two weeks (*n =* 9) (right) lymphomas over days. **(E)** *In vivo* bioluminescence imaging quantification represented as Flux of transplanted DDT-defective (*n =* 9) lymphomas. **(F)** *In vivo* bioluminescence imaging quantification represented as flux of transplanted DDT-defective (*n =* 10) lymphomas in response to 6 mg/kg CsPt treatment every two weeks. Each colored line represents a different mouse. Mice have been grouped in different graphs on the basis of the response to CsPt.

Our previous studies indicated that *Pcna*^*K164R/K164R*^ homozygous mutant primary pre-B cells, as well as *Pcna*^*K164R/K164R*^ homozygous mutant MEFs immortalized by *Trp53* knock down are very sensitive to CsPt, while wild type and heterozygous *Pcna*^*K164R/K164*^ were relatively insensitive [20, 21]. To determine, if a defect in the DDT system enlarges the therapeutic window of tumors to platinums, we generated a cohort of *Trp53*^*–/–*^; *Pcna*^*flox/K164R*^ mice by intercrossing *Trp53*^*–/–*^ with *Pcna*^*flox/K164R*^ mice. As expected, after a short latency period of about 40 days all TP53 deficient mice developed spontaneous lymphomas. To establish an isogeneic DDT-proficient (DDT^P^) and -defective (DDT^D^) lymphoma model we first established a stable cell line from a spontaneous thymic lymphoma that developed in a *Trp53*^*–/–*^; *Pcna*^*flox/K164R*^ mouse. To enable a non-invasive tumor follow-up upon transplantation, we expressed a firefly luciferase by retroviral transduction, using YFP as reporter. Subsequent CRE-mediated deletion of the wild type PCNA^flox^ allele generated a unique set of DDT^P^ and DDT^D^ *Trp53*^*–/–*^ lymphomas (Figure 2A).

### Assessing the *in vitro* CsPt sensitivity of DDT^P^ and DDT^D^ T cell lymphoma

Having established a genetically defined set of DDT^P^ and DDT^D^ lymphoma, we determined their CsPt sensitivity *in vitro*. Independent clones of DDT^P^ and DDT^D^ lymphoma were exposed to increasing doses of CsPt. Three days later, the percentage of viable cells was determined by flow cytometry. Compared to their isogeneic DDT^P^ controls, DDT^D^ clones were hypersensitive to CsPt. Specifically, in the DDT defective condition, the LD_50,_ i.e. CsPt concentration that kills 50 % of lymphoma cells, decreased eight-fold. As independent clones displayed similar CsPt sensitivities, inter-clonal variations affecting CsPt survival could be excluded (Figure 2B) and one clone for each genotype was used for further *in vivo* studies.

### DDT-defective lymphomas are hypersensitive to the maximum-tolerable dose of CsPt

To translate the CsPt sensitivity to an *in vivo* approach, lymphoma cells were transplanted by intravenous injection of one million cells into C57Bl/6BrdCrHsd-Tyr<c> mice. These mice carry an inactivating mutation in the tyrosinase coding gene which prevents hair pigmentation and drastically reduces quenching of bioluminescent signals from the lymphoma. Having stably introduced a luciferase-IRES-YFP construct (*see* Material and Methods) the substrate luciferin was injected intraperitoneally, shortly before measuring tumor growth by *in vivo* imaging system (IVIS) (Figure 2C). Without CsPt treatment, both DDT^P^ and DDT^D^ lymphoma grew aggressively and regardless of the DDT status all recipients had to be euthanized within three weeks after transplantation (Figure 2D left-E).

**Figure 3:**
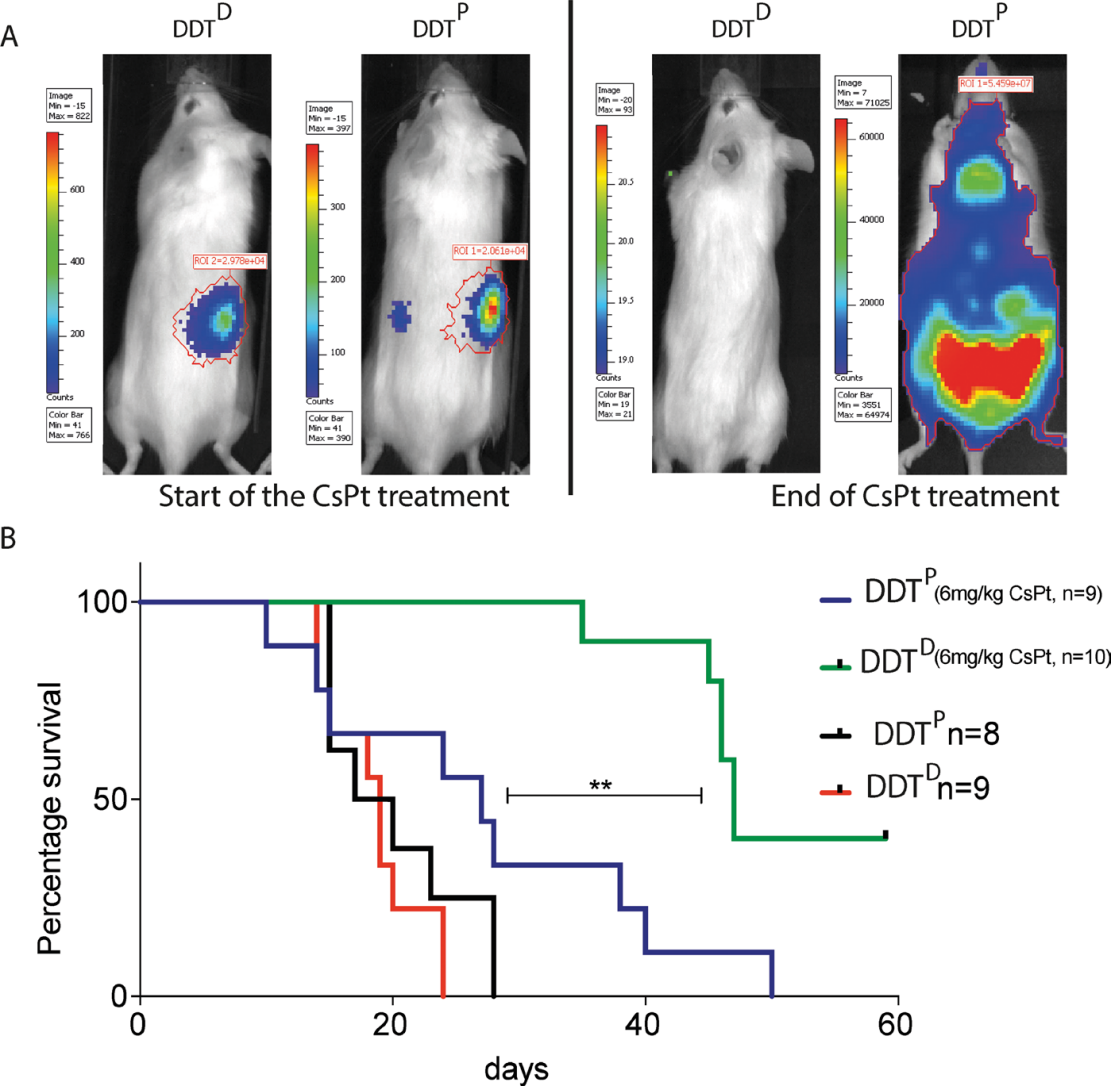
A *Pcna*^*K164R*^ mutation renders lymphomas highly sensitive to CsPt treatment **(A)** Representative examples of DDT^P^ or DDT^D^ tumor bearing mice at the beginning (right), and at the end of the CsPt treatment (left). **(B)** Kaplan – Meier curve of mice carrying DDT^P^ or DDT^D^ tumors. ^**^p = 0.0011 by Mantel-Cox test when comparing DDT^P^ or DDT^D^ tumor bearing mice treated with CsPt. Treated groups received 6 mg/kg CsPt every two weeks, starting from the first bioluminescent signal detection by IVIS.

To determine the *in vivo* sensitivity of DDT^P^ and DDT^D^ lymphoma to CsPt we administered the maximum-tolerable dose (MTD) 6 mg/kg CsPt dose [22] intravenously, to DDT^P^ and DDT^D^ lymphoma bearing mice. The CsPt administration was started as soon as the first tumor bioluminescent signal was detected, and beyond this time point the tumor growth was followed weekly. While CsPt treatment could slightly delay tumor growth in the DDT^P^ setting (Figure 2D right), it was highly effective in treating DDT^D^ lymphoma, as depicted in the three panels of Figure 2F. In 8 out of 10 mice, a single CsPt administration eradicated most of the tumor mass, as measured by the IVIS (Figures 2F, 3A). Repetitive treatments could control the DDT^D^ tumor load effectively but since the lymphoma reappeared, CsPt was apparently insufficient to eradicate the entire tumor (Figure 3B).

As apparent from the Kaplan–Meier plot, treatment of DDT^P^ lymphoma mice with 6 mg/kg CsPt slightly but not significantly increased tumor survival. In contrast, 40% of mice bearing the DDT^D^ lymphoma survived without a detectable tumor up to 59 days and a significant increase in survival was gained when compared to CsPt-treated DDT^P^ lymphoma reference (Figure 3B). However, 3 out of 9 mice in the DDT^D^ setting had to be euthanized because of brain metastasis. Apparently, some lymphoma cells traversed the blood brain barrier and, given the poor accessibility of CsPt to the brain, this provided an ideal niche to escape the otherwise systemic CsPt pressure (Supplementary Figure 1A).

### DDT-defective lymphomas are highly sensitive to low CsPt regime

**Figure 4:**
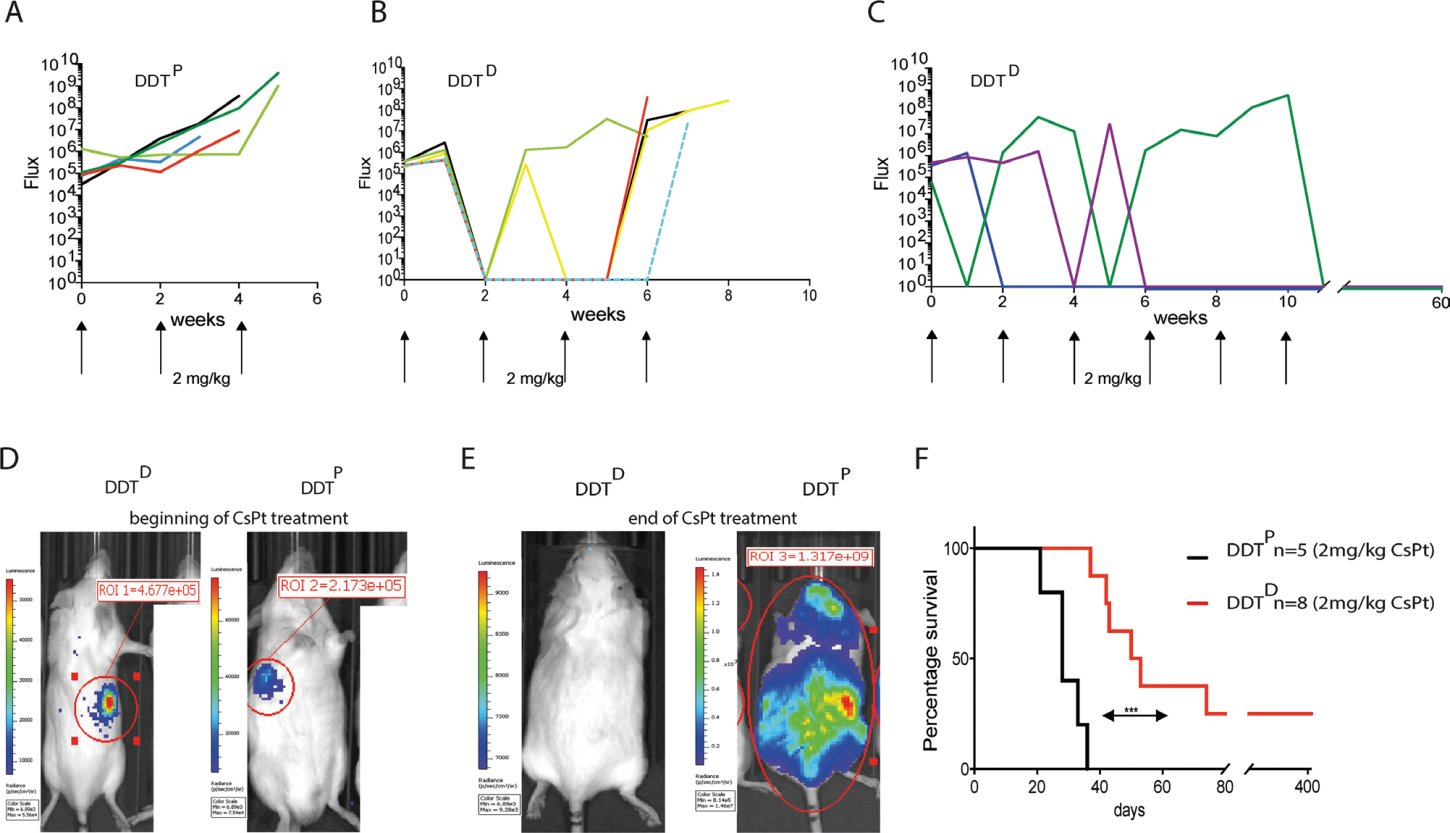
Low dose CsPt therapy is effective in DDT-defective lymphomas **(A)** Curve represents the flux of bioluminescence signal from IVIS detection of mice (*n =* 5) carrying WT tumor in response to 2 mg/kg CsPt treatment every two weeks. Each line represents an indicated mouse. **(B–C)** As in A but DDT^D^ lymphoma (*n =* 8) readouts are displayed. **(D)** Representative examples of DDT^P^ or DDT^D^ lymphoma bearing mice at the beginning of the 2 mg/kg CsPt treatment, maintained every two weeks. **(E)** As in **(D)** but at the end of the CsPt regime. **(F)** Kaplan–Meier curve of mice carrying tumor with indicated genotypes upon 2 mg/kg CsPt treatment. ^***^p = 0.0001 by Mantel Cox test.

Despite the effectiveness of CsPt towards many cancer types, toxicities associated with CsPt are a major clinical problem and pharmacological challenge. Toxicities range from mild to severe, with peripheral neurotoxicity and especially nephrotoxicity being the most serious [23]. Given these limitations associated with standard CsPt treatment and the high sensitivity of DDT^D^ lymphoma to low concentrations of CsPt *in vitro*, we predicted that tumor-specific defects in PCNA ubiquitination could actually chemosensitize those tumors to lower doses of CsPt *in vivo*. Using 2 mg/kg CsPt, a 3-fold lower dose than the MTD, the DDT^P^ lymphoma remained as expected relatively unresponsive to the treatment, and recipients carrying this tumor needed to be sacrificed within 40 days after initial tumor detection (Figure 4A, 4D, 4E). In contrast, all the mice bearing DDT^D^ lymphoma responded very well to the initial low dose therapy (Figure 4D, 4E). 75% of mice in DDT^D^ defective setting survived from 6 to 11 weeks after the initial tumor detection (Figure 4B–4E). The mouse treated up to 11 weeks needed to be sacrificed because of body weight loss but was tumor free at the moment of necropsy. Most remarkably, 25% of mice could be considered entirely tumor free as measured by IVIS readouts and survived 420 days (Figure 4F).

### Modification of *Wap–Cre;Cdh1*^*F/F*^*;SB* mammary gland tumor and assessment of *in vitro* CsPt sensitivity

**Figure 5:**
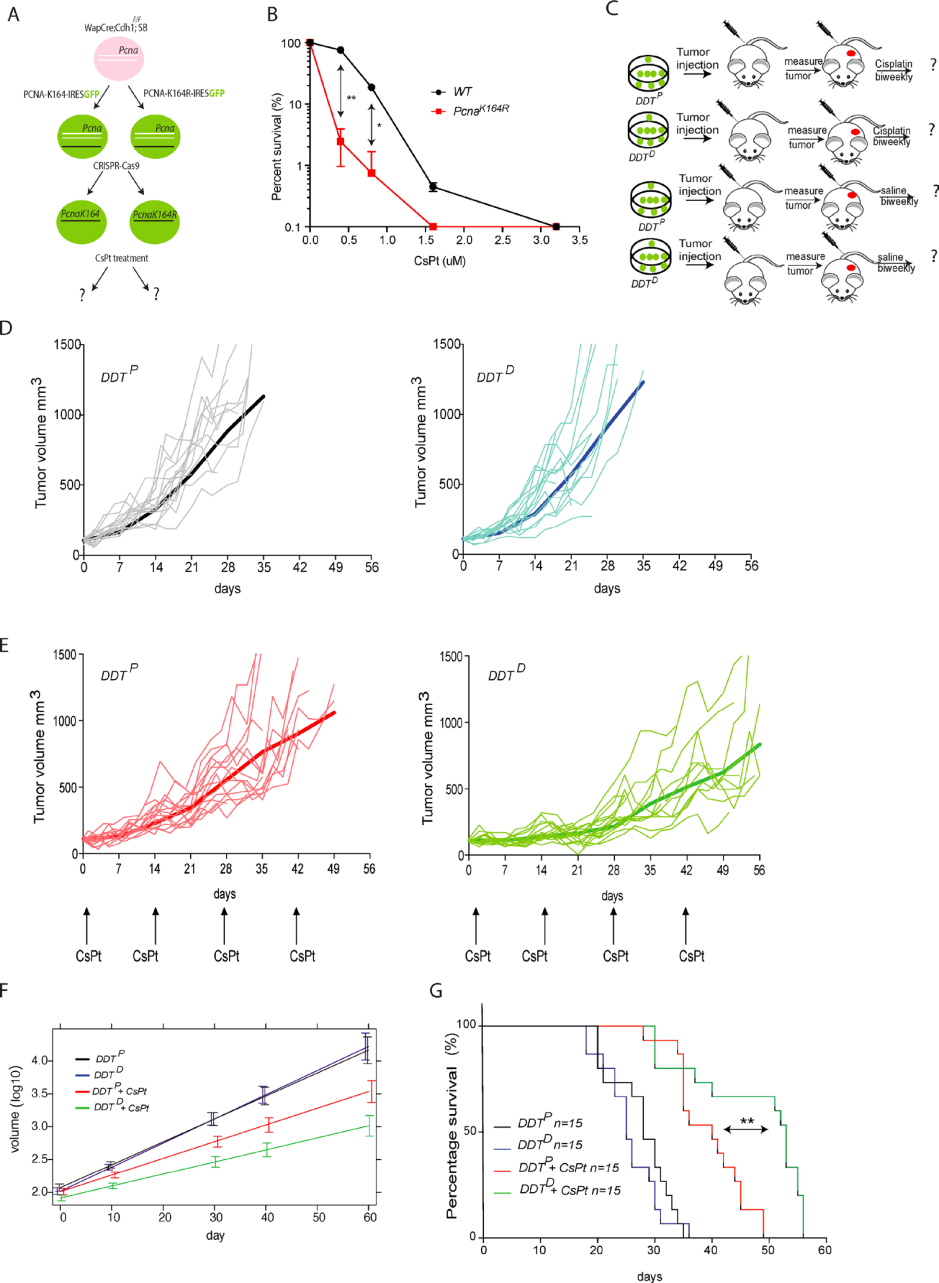
Assessing the chemosensitizing potential of a DDT blockade in mammary gland tumor model **(A)** Graphic representation of *in vitro* modification of mammary gland tumor cells to derive *DDT*^*P*^ and *DDT*^*D*^ invasive lobular breast carcinoma lines. **(B)** Cell survival in response to different concentration of CsPt. Mammary tumor cell survival was normalized to the mock-treated cells for each condition. Unpaired *t* test with Welch’s correction ^**^P < 0.01; ^*^P < 0.5 was used to check the significance. The average of two independent experiments with two independent cell lines per genotype is plotted ± SD. **(C)** Schematic representation of mammary gland tumor cells transplantation and follow-up. *Wap–Cre;Cdh1*^*F/F*^*; SB;Pcna*^*K164*^ (DDT^P^) and *Wap–Cre;Cdh1*^*F/F*^*; SB;Pcna*^*K146R*^ (DDT^D^) tumor cells were transplanted in the fourth mammary fat pad of nude (NMRI) recipient mice. Tumor growth was monitored three times per week to assess treatment efficacy. Each experimental group contains 15 mice. **(E)** As in **(D)**, but here mice where enrolled in the treatment schedule when tumors had reached a size of 100 mm^3^. Mice were treated with MTD 6 mg/kg CsPt every two weeks. **(F)** Colored curves represent the average trajectory in each group over time of the tumor volumes measured on a logarithmic scale according to a linear mixed-effect model fitted by REML. Comparison between treated DDT^P^ and treated DDT^D^ gives a *p* < 0.001. **(G)** Kaplan–Meier curve of treated vs non-treated tumor bearing mice of indicated genotype. ^**^p = 0.0012 according to Mantel-Cox test, when comparing survival of mice with a DDT^P^ or DDT^D^ mammary gland tumor, upon 6 mg/kg CsPt treatment, administered every two weeks.

To extend our findings to an independent highly metastatic tumor model, we made use of *Wap-Cre;Cdh1*^*F/F*^*; SB* invasive lobular carcinoma cell line established by Kas *et al.* [24]. In order to obtain DDT^P^ and DDT^D^ mammary gland tumors, we modified the above-mentioned tumor cells *in vitro*. First, we expressed a wild type PCNA or mutant PCNA^K164R^ cDNA by stable transduction. Subsequently, we deleted the endogenous PCNA WT alleles via CRISPR/Cas9 mediated approach (Figure 5A). In this way two isogenic *Wap-Cre;Cdh1*^*F/F*^*;SB;Pcna*^*K164*^ (DDT^P^) and *Wap-Cre;Cdh1*^*F/F*^*;SB;Pcna*^*K146R*^ (DDT^D^) mammary tumor cell lines were established. Having established this unique isogeneic set of DDT^P^ and DDT^D^ invasive lobular breast carcinoma, we first assessed their proliferation capacity and observed no difference in growth between the two (Supplementary Figure 3). Subsequently, we tested their sensitivity to increasing dose of CsPt *in vitro*. Compared to DDT^P^, the DDT^D^ mammary tumor was highly sensitive to low CsPt doses (Figure 5B), which corroborated our findings in the lymphoma set up.

### *In vivo* responsiveness of DDT^P^ and DDT^D^ mammary tumors to CsPt

To evaluate the chemosensitizing potential of *Pcna*^*K146R*^ versus *Pcna*^*K164*^ mammary tumor cells we transplanted tumor cells orthotopically into the 4th fat pad of the mammary gland. When tumors reached 100mm^3^, usually within 2 weeks after transplantation, mice were enrolled in the treatment group or the control group (Figure 5C). *In vivo* tumor growth of wild type and mutant cell lines was comparable. All the mice enrolled in the mock treatment groups reached the humane end point within 35 days and were euthanized (Figure 5D). Given the aggressiveness and metastatic potential of these cell lines, mice often had to be sacrificed before reaching the tumor size of 1500 mm^3^ at the primary site of transplantation. While only 13% of the mice with DDT^P^ tumors could benefit from the treatment up to 49 days, 67% of the mice with a DDT^D^ tumor survived more than 50 days (Figure 5E–5G). In detail, comparing the different growth curves (Figure 5F) it was evident that the *Pcna*^*K164R*^ tumor cells were controlled in the first 28 days of the CsPt treatment, but they quickly became unresponsive beyond this time window. In summary, although the mice carrying a *Wap–Cre;Cdh1*^*F/F*^*;SB;Pcna*^*K164R*^ mammary gland tumor could not be cured, the tumor outgrowth was significantly delayed by this monotherapy (Figure 5G).

## DISCUSSION

Tumors with specific defects in the DDR network offer great potential for intervention with defined DNA damaging agents that selectively target this vulnerability. The fact that TLS constitutes an essential intermediate step in ICL repair, strongly motivated us to further explore this therapeutic tactic. A survey of TCGA revealed a high frequency of human tumors with predicted, homozygous inactivating deletions of genes coding components of the DDT network. Of note, analysis of TCGA showed that 11,2% of renal cell carcinoma (RCC) and ∼5% of pancreatic tumors have inactivating *RAD18*-deletions and 6,7% of malignant peripheral nerve sheath tumors lack *RAD6B*. As each TLS polymerase tolerates preferential types of lesions [14], DDT defects are predicted to render tumors hypersensitive to specific lesions that can be inflicted by many endogenous and exogenous DNA damaging agents, including clinically approved and widely applied DNA crosslinking anti-cancer drugs. Given the high frequency of homozygous inactivating deletions for key players in the DDT system (Figure 1) and rapid advancements in NGS based cancer diagnostics, there is an unmet need to screen for tumor-specific vulnerabilities in the DDT system. As alternative PCNA-K164 E2/E3 ubiquitination systems exist, their functional (non)redundancy in different cell types remains to be determined [7, 25, 26].

We have previously reported that *Pcna*^*K164R/K164R*^ mutant cells and mice are strongly impaired in DDT and consequently highly sensitive to replication blocking lesions [21, 20]. These observations and the site-specificity of the PCNA-ubiquitination reaction imply that tumor-specific DDT defects may widen the therapeutic window for alkylating and platinating agents and provide a unique opportunity to sensitize selectively these tumors to platinum-based therapies, limit toxicities, and improve the overall therapeutic outcome. Using well defined genetically engineered mouse models and cell lines, the present study defines the potential therapeutic gain for tumors defective in PCNA-ubiquitination promoted DDT. The ultimate goal of this bench to bedside research, is to delineate the therapeutic importance of screening cancers for specific DDT defects, define their specific vulnerabilities and therapeutic windows, in order to optimize cancer therapy.

To test our hypothesis, we combined our established genetically engineered mouse models with CRISPR/Cas9 technology to derive PCNA^K164^ proficient or defective tumors. The *in vitro* hypersensitivity of mutant, i.e. DDT^D^ tumors to CsPt predicted a high responsiveness and warranted further studies *in vivo*. Testing the efficacy of CsPt on DDT-proficient and -defective lymphoma, only mice carrying the DDT-defective lymphoma could be treated effectively, and responded for long term to this monotherapy. In fact, already a single dose was capable to reduce the bioluminescent signal selectively in the DDT^D^ condition, while the DDT^P^ tumors continued their growth and remained largely unresponsive. This study proofs that *Trp53*^*–/–*^ lymphomas lacking PCNA^K164^-facilitated DDT not only are highly sensitive to low dose chemotherapy, but also that *in vivo* CsPt treatment can cure 25% of mice suffering from a DDT^D^ lymphoma. This exciting result is quite remarkable, considering that monotherapy with a single platinating agent is often ineffective in long term and needs to be interrupted because of tumor unresponsiveness or toxicities. Our data clearly suggests that a DDT defect in a metastatic and aggressive tumor type can sensitize the tumor to conventional CsPt treatment.

To determine independently if the findings made in the lymphoma model could be extended to a solid tumor model with a different genetic and phenotypic background, we chose for a highly metastatic tumor cell line established from the Wap–Cre; Cdh1^F/F^;SB system. Given the difficulty to selectively kill solid tumors by mono-chemotherapy, a DDT defect might be a good indication for adjuvant agents that target specifically this class of DDR defects. Having established an isogenic set of a DDT^P^ and DDT^D^ invasive lobular carcinomas by CRISPR/Cas9 approach, we here tested their therapeutic potential to CsPt based therapy. Comparing the different individual mice as well as cumulative lines, it emerged that only mice carrying the mutant tumors had a significant benefit following this monotherapy, while at the same time the DDT^P^ carcinoma remained not majorly affected. Apparently, the treatment of invasive lobular carcinoma can benefit from adjuvant therapies that take advantage of specific DDT defects. The fact that after an initial growth perturbation, the DDT^D^ tumor quickly adapts and escapes this selective pressure, suggests the existence of an effective, alternative DDT pathways capable of recruiting and activating TLS polymerases to replication blocking lesions, including unhooked ICLs. Alternatively, tumor intrinsic resistance mechanisms may affect the CsPt sensitivity of a tumor. The different responsiveness of lymphoma and breast carcinoma to CsPt monotherapy likely relate to differential capacities of specific cell types to switch between alternative TLS activation modalities. Screening for synthetic lethality might help to discover new combinational therapies. These therapies might be the key to tackle the limitations of current pharmacological approaches.

Taken together, our data revealed that tumors holes in the DDT network, and especially in RAD6/RAD18 pathway, can indicate vulnerabilities that enlarge therapeutic windows, and offer unique opportunities to optimize tumor-specific intervention with drugs that impinge on tumor weakness. This requires, besides well-established approved chemotherapeutics, the development of new drugs that selectively target tumor-specific DDT defects and achieve synthetic lethality in the DDT system.

Cancer intervention with CsPt in the DDT-defective tumor context, can offer higher cure rates with less side effects and provide a better quality of life for this patient group.

## MATERIALS AND METHODS

### Generation of *Trp53*^–/–^;*Pcna*^*Flox/K164R*^ mice. In order to obtain spontaneous tumor we crossed *Pcna*^*Flox/K164R*^ mice [27] with the tumor prone strain *Trp53*^*–/–*^ [28]

#### *In vitro* culture, modification of *Trp53*^*–/–*^*;Pcna*^*Flox/K164R*^ tumor and *In vitro* Cisplatin sensitivity test

After *Trp53*^*–/–*^*;Pcna*^*Flox/K164R*^ mice developed tumors, the mouse was sacrificed by CO_2_ inhalation and the affected lymphoid organs were isolated. Single cell suspension was made by mechanical meshing and filtering through a 70 um filter. Different cell concentrations were used to start the primary culture of lymphoma cells using RPMI medium supplemented with 8% fetal calf serum (FCS), 100 µM pen/strep, 100 µM β-mercaptoethanol, and 200 µM of L-Asparagine. Cells were cultured under standard culturing conditions.

The CRE mediated deletion of the floxed allele was obtained by transducing the tumor cells with the retrovirus pMSCV-Cre-ERT2-Blasticidine. The cells were selected for Blasticidine resistance for 3 days with medium containing 1 ug/ml of Blasticidine S-HCl (R21001, Life technologies), and 1 µM of 4-hydroxytamoxifen (Sigma-Aldrich Chemie B.V.) was used to induce the CRE mediate deletion of the floxed allele. A specific PCR strategy was applied to assess the deletion of floxed allele (Supplementary Table 1).

To check *in vitro* sensitivity against CsPt, 1 × 10^5^ lymphoma cells were seeded in 24-wells plates in 1 ml complete medium containing different concentrations of CsPt. To determine cell survival, cells kept under condition with or without CsPt, were harvested after 3 days of culture and stained with propidium iodine (PI). The number of PI-negative cells was measured on a FACSArray (Becton Dickinson). Data analysis was performed with FlowJo software.

### Transplantation, *in vivo* bioluminescence imaging and CsPt treatment of lymphoma model

1 × 10^6^ cells were resuspended in sterile PBS and injected intravenously (i.v.). Mice were monitored for luminescence signal twice or once a week by *In Vivo* Imagining system (IVIS) before or after tumor development, respectively. Beetle luciferin (Promega) was dissolved at 15 mg/mL in sterile PBS solution and stored at −20° C. Luciferin solution was injected intraperitoneally (i.p.) (10 µg/L mL/g body weight) and animals were anesthetized with 2–3% isoflurane. Light emission was measured 15 min after luciferin administration by using a cooled CCD camera (IVIS; Xenogen), coupled to Living Image acquisition and analysis software over an integration time of 1 min. Signal intensity was quantified as the Flux (photons per second) measured over selected area of interest. The mice were randomly selected to start CsPt treatment as soon as the first bioluminescent signal was detected. 2 or 6 mg/kg of CsPt was administered per mouse with intervals of two weeks, with a maximum of six injections per mouse.

### *In vitro* modification of *Wap–Cre;Cdh1F/F;SB tumor cells*

*Wap–Cre;Cdh1F/F;SB* tumor cells resemble the human invasive lobular carcinoma. Details regarding the generation of this genetically engineered mouse model have been described previously [24]*. Wap–Cre;Cdh1F/F;SB* cells are fast adherent growing cells. The established tumor cell line was cultured under standard conditions in RPMI medium supplemented with 8% FCS, 100 µM pen/strep and 100 µM β-mercaptoethanol. To stably transduce the tumors cells with a *PcnaK164* or *PcnaK164R cDNA,* we followed this procedure. 5^*^ × 10^5^ of HEK293T cells were seeded in a 6-well plate with 2 ml complete medium per well (IMDM, supplemented with 8% fetal calf serum (FCS), pen/stre (100 µM), and 2-mercaptoethanol (100 µM), and 2-mercaptoethanol (100 µM)) and cultured under standard conditions. The following day, HEK293T cells were transfected with 6 µl X-tremeGENE (Roche), 194 µl of serum free medium (SFM) and incubated for 5 min at RT. 2 µg of pMX-IRES-GFP-PCNA^WT^ or -PCNA^K164R^ and packaging vector (pCL-Eco) were added in a total of 200 µl Serum Free Medium. The ratio of X-tremeGENE to total DNA was 3:1 while that for the plasmid to pCL-Eco was 3:2. Both X-tremeGENE and plasmid mix were put together and incubated for 30 min at RT. Following incubation, 400 µl of the final mixture was added dropwise to each well already containing 1,6 ml of complete IMDM medium. These cells were cultured under standard conditions for 48 hr after which the supernatant containing retroviral particles was collected. To improve the transduction efficiency, Polybrene^®^ (10 mg/ml) was added to the supernatant at a final concentration of 1 mg/ml. 1 ml of virus supernatant was added to 0.5 ml tumor cells. After 48 hours post transduction, tumor cells were visually examined for GFP+ expression.

To delete the endogenous PCNA alleles in transduced (GFP+) cells, the CRISPR/Cas9 system was used. Initial design of *pX333* was modified by putting mCherry along with T2A sequence downstream of Cas9. This enabled us to sort mCherry positive cells that ensured the transfection of both gRNAs. gRNAs targeting intronic regions flanking exon 2 and 4 only and not the PCNA cDNA were cloned into *pX333* (gRNAs sequence in Supplementary Table 2A). For transfecting the breast cancer cells with *pX333* carrying two gRNAs, the optimal ratio of 8:2 of FuGENE^®^ 6 (µl): *pX333* (µg) was used in a total volume of 200 µl of SFM. Following 24 hours post transfection, cells were sorted as single clones based on mCherry expression.

A PCR strategy with several primers flanking exon 2 and 4 was designed to check the deletion of endogenous PCNA allele in sorted single clones (Supplementary Figure 2A and Supplementary Table 2B), (Supplementary Table 3). Biallelic disruption of endogenous PCNA allele was further confirmed using a set of internal primers (Supplementary Table 3, Supplementary Figure 2A). The clones that carried the deletion were selected for further analysis.

### Colony survival of mammary gland tumor cells

*Wap–Cre; Cdh1F/F;SB* tumor cells were seeded in 10 cm dishes in complete medium with varying cell concentrations. One day later, the medium was replaced with complete medium containing the indicated concentrations of CsPt. Eight days later cells were washed with PBS and fixed in 5 ml of methanol : acetic acid (3:1) for 1 h. Colonies were stained by adding 3 ml of 0.3% Coomassie Brilliant Blue solution. After 1 h, the dished were washed with H_2_O and allowed to dry and colonies were counted. Survival of CsPt-treated cells was corrected for the plating efficiency of the untreated cells. Data points represent the mean survival relative to the untreated control cells.

### Tumor transplantation and *in vivo* treatment

2 × 10^5^ *Wap–Cre; Cdh1F/F;SB* mammary tumors cells DDT^P^ or DDT^D^ mutant *were* resuspended in PBS and Matrigel 1:1 and transplanted orthotopically into the fourth right mammary fat pad of NMRI mice as described by NKI standard operating procedure (SOP). NMRI mice were used to exclude potential immune reactions, because the tumors were of mixed FVB/C57BL/6 background. CsPt treatment (6 mg/kg of CsPt (i.v.) per mouse with intervals of two weeks, with a maximum of six injections per mouse) was started when tumors reached a size of ∼100 mm^3^ (formula for tumor volume: 0.5 × length × width) and mice were monitored three times a week. Mice were killed either when the tumor volume exceeded 1,500 mm^3^ or when it metastasized and caused severe overall distress to the mouse.

### Statistical analysis

To assess the statistical significance of our data we used *t*–test or Mantel-Cox test (^*^P < 0.05, ^**^P < 0.01, ^***^P < 0.001,) performed by Prism 7 (GraphPad).

For the Figure 5F we compared the tumor growth trajectories by applying a linear mixed-effects model fitted by REML. The outcome was a tumor volume measured on a logarithmic scale and the factors were condition, genotype and condition*genotype interaction. As output of this model we found that the average growth of tumor volume was different for the 2 genotypes under treatment (interaction effect = –0.007, *p*-value < 0.001).

## SUPPLEMENTARY MATERIALS FIGURES AND TABLES



## Abbreviations

CsPt: Cisplatin
DDR: DNA damage response
DDT: DNA damage tolerance
DSBs: DNA double strand breaks
ICL: Interstrand Crosslink
NGS: Next-Generation-Sequencing
POL: Polymerase
TCGA: The Cancer Genome Atlas
TLS: Translesion Synthesis

## References

[1] Farmer H, McCabe N, Lord CJ, Tutt AN, Johnson DA, Richardson TB, Santarosa M, Dillon KJ, Hickson I, Knights C, Martin NM, Jackson SP, Smith GC (2005). Targeting the DNA repair defect in BRCA mutant cells as a therapeutic strategy. Nature.

[2] Jackson SP, Bartek J (2009). The DNA-damage response in human biology and disease. Nature.

[3] Friedberg EC (2005). Suffering in silence: the tolerance of DNA damage. Nat Rev Mol Cell Biol.

[4] Moldovan GL, Pfander B, Jentsch S (2007). PCNA, the maestro of the replication fork. Cell.

[5] Stelter P, Ulrich HD (2003). Control of spontaneous and damage-induced mutagenesis by SUMO and ubiquitin conjugation. Nature.

[6] Vujanovic M, Krietsch J, Raso MC, Terraneo N, Zellweger R, Schmid JA, Taglialatela A, Huang JW, Holland CL, Zwicky K, Herrador R, Jacobs H, Cortez D (2017). Replication Fork Slowing and Reversal upon DNA Damage Require PCNA Polyubiquitination and ZRANB3 DNA Translocase Activity. Mol Cell.

[7] Hoege C, Pfander B, Moldovan GL, Pyrowolakis G, Jentsch S (2002). RAD6-dependent DNA repair is linked to modification of PCNA by ubiquitin and SUMO. Nature.

[8] Haracska L, Torres-Ramos CA, Johnson RE, Prakash S, Prakash L (2004). Opposing effects of ubiquitin conjugation and SUMO modification of PCNA on replicational bypass of DNA lesions in Saccharomyces cerevisiae. Mol Cell Biol.

[9] Kannouche PL, Wing J, Lehmann AR (2004). Interaction of human DNA polymerase eta with monoubiquitinated PCNA: a possible mechanism for the polymerase switch in response to DNA damage. Mol Cell.

[10] Bienko M, Green CM, Crosetto N, Rudolf F, Zapart G, Coull B, Kannouche P, Wider G, Peter M, Lehmann AR, Hofmann K, Dikic I (2005). Ubiquitin-binding domains in Y-family polymerases regulate translesion synthesis. Science.

[11] Ulrich HD (2004). How to activate a damage-tolerant polymerase: consequences of PCNA modifications by ubiquitin and SUMO. Cell Cycle.

[12] McCulloch SD, Kunkel TA (2008). The fidelity of DNA synthesis by eukaryotic replicative and translesion synthesis polymerases. Cell Res.

[13] Waters LS, Minesinger BK, Wiltrout ME, D’Souza S, Woodruff RV, Walker GC (2009). Eukaryotic translesion polymerases and their roles and regulation in DNA damage tolerance. Microbiol Mol Biol Rev.

[14] Prakash S, Johnson RE, Prakash L (2005). Eukaryotic translesion synthesis DNA polymerases: specificity of structure and function. Annu Rev Biochem.

[15] Deans AJ, West SC (2011). DNA interstrand crosslink repair and cancer. Nat Rev Cancer.

[16] Pilzecker B, Buoninfante OA, Pritchard C, Blomberg OS, Huijbers IJ, van den Berk PC, Jacobs H (2016). PrimPol prevents APOBEC/AID family mediated DNA mutagenesis. Nucleic Acids Res.

[17] Gao J, Aksoy BA, Dogrusoz U, Dresdner G, Gross B, Sumer SO, Sun Y, Jacobsen A, Sinha R, Larsson E, Cerami E, Sander C, Schultz N (2013). Integrative analysis of complex cancer genomics and clinical profiles using the cBioPortal. Sci Signal.

[18] Cerami E, Gao J, Dogrusoz U, Gross BE, Sumer SO, Aksoy BA, Jacobsen A, Byrne CJ, Heuer ML, Larsson E, Antipin Y, Reva B, Goldberg AP (2012). The cBio cancer genomics portal: an open platform for exploring multidimensional cancer genomics data. Cancer Discov.

[19] Acharya N, Haracska L, Prakash S, Prakash L (2007). Complex formation of yeast Rev1 with DNA polymerase eta. Mol Cell Biol.

[20] Pilzecker B, Buoninfante OA, van den Berk P, Lancini C, Song JY, Citterio E, Jacobs H (2017). DNA damage tolerance in hematopoietic stem and progenitor cells in mice. Proc Natl Acad Sci U S A.

[21] Langerak P, Nygren AO, Krijger PH, van den Berk PC, Jacobs H (2007). A/T mutagenesis in hypermutated immunoglobulin genes strongly depends on PCNAK164 modification. J Exp Med.

[22] Rottenberg S, Nygren AO, Pajic M, van Leeuwen FW, van der Heijden I, van de Wetering K, Liu X, de Visser KE, Gilhuijs KG, van Tellingen O, Schouten JP, Jonkers J, Borst P (2007). Selective induction of chemotherapy resistance of mammary tumors in a conditional mouse model for hereditary breast cancer. Proc Natl Acad Sci U S A.

[23] Dasari S, Tchounwou PB (2014). Cisplatin in cancer therapy: molecular mechanisms of action. Eur J Pharmacol.

[24] Kas SM, de Ruiter JR, Schipper K, Annunziato S, Schut E, Klarenbeek S, Drenth AP, van der Burg E, Klijn C, Ten Hoeve JJ, Adams DJ, Koudijs MJ, Wesseling J (2017). Insertional mutagenesis identifies drivers of a novel oncogenic pathway in invasive lobular breast carcinoma. Nat Genet.

[25] Broomfield S, Chow BL, Xiao W (1998). MMS2, encoding a ubiquitin-conjugating-enzyme-like protein, is a member of the yeast error-free postreplication repair pathway. Proc Natl Acad Sci U S A.

[26] Ulrich HD, Jentsch S (2000). Two RING finger proteins mediate cooperation between ubiquitin-conjugating enzymes in DNA repair. EMBO J.

[27] Langerak P, Nygren AO, Schouten JP, Jacobs H (2005). Rapid and quantitative detection of homologous and non-homologous recombination events using three oligonucleotide MLPA. Nucleic Acids Res.

[28] Donehower LA, Harvey M, Slagle BL, McArthur MJ, Montgomery CA, Butel JS, Bradley A (1992). Mice deficient for p53 are developmentally normal but susceptible to spontaneous tumours. Nature.

