# Supplementary material for: Precision cancer therapy: profiting from tumor specific defects in the DNA damage tolerance system
Source: Oncotarget. 2018 Apr 10;9(27):18832–43. doi: 10.18632/oncotarget.24777 (PMC5922359; doi:10.18632/oncotarget.24777)
Supplement: Supplementary file 1 [file oncotarget-09-18832-s001.pdf]

Precision cancer therapy: profiting from tumor specific defects in the DNA damage tolerance system

SUPPLEMENTARY MATERIALS

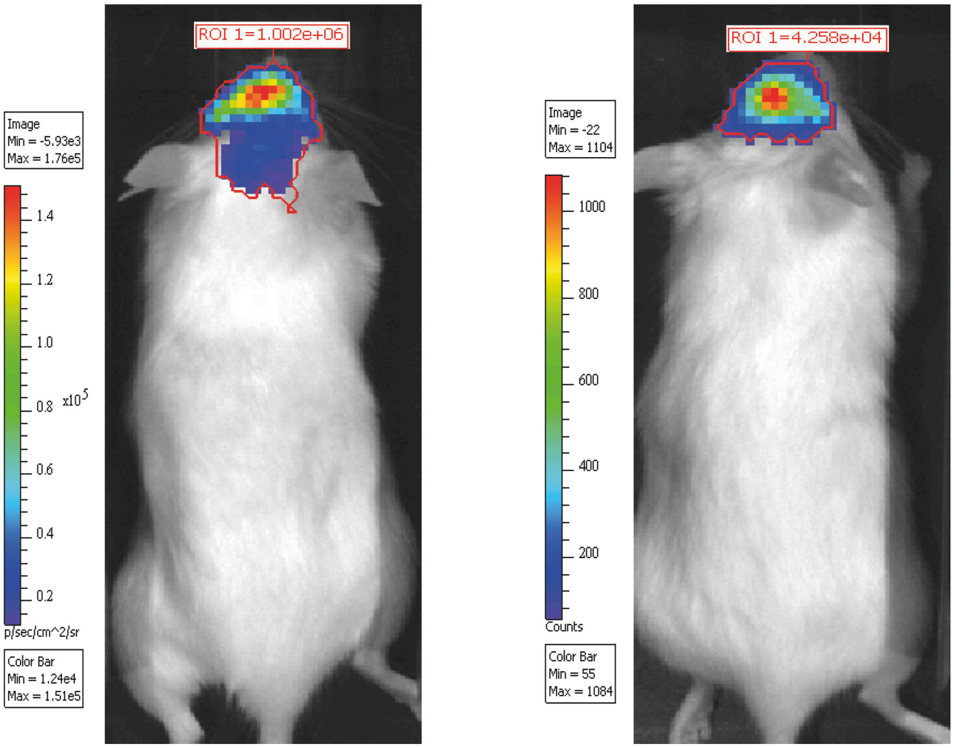

Supplementary Figure 1: (**A**) Representative IVIS images of CsPt treated mice carrying a DDTD tumor. Brain tumor developed after consecutive CsPt treatments.

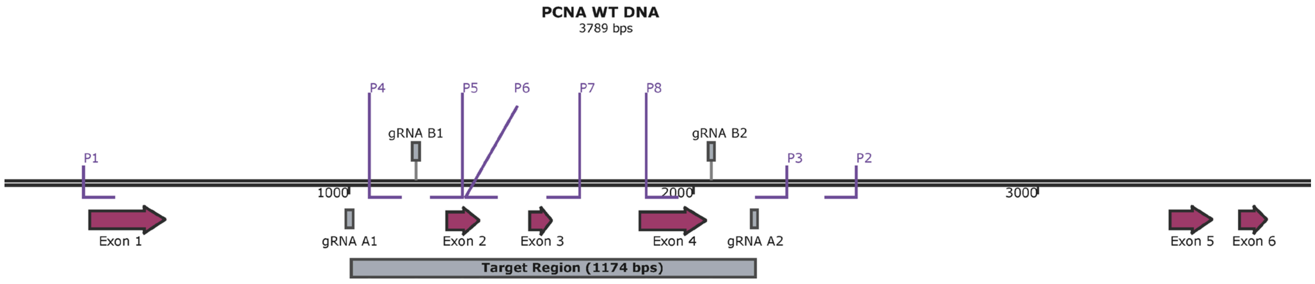

Supplementary Figure 2: (**A**) Targeting strategy of endogenous PCNA WT allele. Two pairs of CRISPR/Cas9 gRNA (A1 + 2 and B1 + 2), targeting the indicated regions, have been used to delete the PCNA alleles in the invasive lobular breast carcinoma cells. The primers used to screen for deletion have been described in Supplementary Table 2–3 and are indicated in the map as P1–P8.

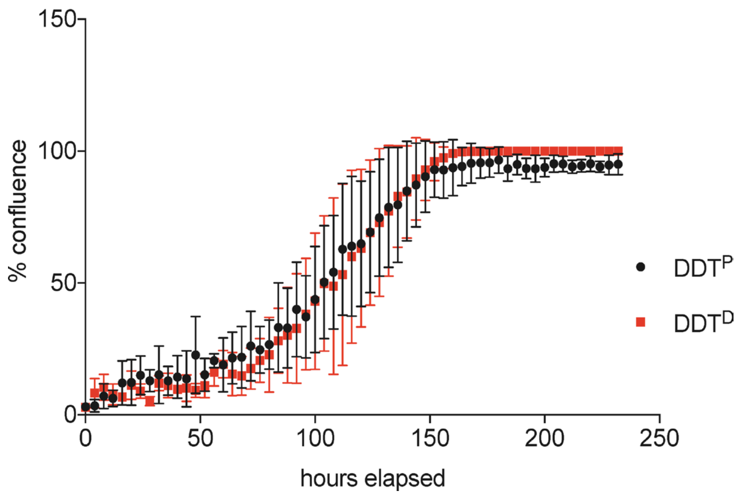

Supplementary Figure 3: (**A**) The graph displays the cell confluence in percentage (%) of DDTP and DDTD breast tumor cells measured using IncuCyte® live-cell imaging. Using 96 well plates, each cell line was seeded in quadruple at a density of 300 cells per well and proliferation was measured on the basis of cell density (IncuCyte®).

Supplementary Table 1: PCR primers to assess the deletion of the floxed PCNA allele

Forward	CCTGAGAGAGTTTGCCTGCAGG
Reverse	CTTTTGCTCACTTGCCAACCCTC

Supplementary Table 2A: Sequences of gRNA's used to target the endogenous PCNA allele

gRNA	(5′….3′)
gRNA-A1	GAAAAGGCCAGACGTGATG
gRNA-A2	GACTCCATAAGCTTTTAC
gRNA-B 1	ACCGTGAGTGTGAAATGGTA
gRNA-B 2	TAGTAAGGGGGCGTCCAGTT

Supplementary Table 2B: Sequences of primers used to screen for deletion

Primers	(5′….3′)
P1	GTCGCCACAACTCCGCCACC
P2	GCTGAGAATCTTCCTGCTGTAGC
P3	GAGGGTTGGCAAGTGAGCAAAAG
P4	GATGCTTTCTCGAATGTTGGCTTTTAG
P5	GTAATGATGTCTTCATTACCAGCAC
P6	GGGCTGAAGATAATGCAGACACC
P7	CACCACTCCACTCAGAACACGCA
P8	CTTTTGCTCACTTGCCAACCCTC
P9*	TTCGCCACCTCTGACTTGAGCGT

*was used to sequence the pX333 for checking the insertion of gRNAs.

Supplementary Table 3: PCR product sizes of the indicated primer pairs

External Primers	WT	Deletion
P1 + P2	2247 Bps	1073 Bps
P1 + P3	2046 Bps	872 Bps
**Internal Primers**	**WT**	**Deleted**
P4 + P5	260 Bps	No Amplification
P6 + P7	334 Bps	No Amplification
P8 + P3	406 Bps	No Amplification
